# Universal Health Coverage for Schizophrenia: A Global Mental Health Priority

**DOI:** 10.1093/schbul/sbv107

**Published:** 2015-08-05

**Authors:** Vikram Patel

**Affiliations:** ^1^London School of Hygiene & Tropical Medicine, the Public Health Foundation of India and Sangath, Goa, India

## Abstract

The growing momentum towards a global consensus on universal health coverage, alongside an acknowledgment of the urgency and importance of a comprehensive mental health action plan, offers a unique opportunity for a substantial scale-up of evidence-based interventions and packages of care for a range of mental disorders in all countries. There is a robust evidence base testifying to the effectiveness of drug and psychosocial interventions for people with schizophrenia and to the feasibility, acceptability and cost-effectiveness of the delivery of these interventions through a collaborative care model in low resource settings. While there are a number of barriers to scaling up this evidence, for eg, the finances needed to train and deploy community based workers and the lack of agency for people with schizophrenia, the experiences of some upper middle income countries show that sustained political commitment, allocation of transitional financial resources to develop community services, a commitment to an integrated approach with a strong role for community based institutions and providers, and a progressive realization of coverage are the key ingredients for scale up of services for schizophrenia.

## Introduction

In September 2015, the United Nations will host a major congress to finalize the shape of the Sustainable Development Goals (SDGs), replacing the Millenium Development Goals agreed in 2000 with a timetable of 15 years. The scope of the SDGs is likely to cover a wide range of concerns, from health and education to conflict and environmental sustainability.^1^ Within the health goal, there are likely to be a range of targets, and there is a growing advocacy and a strong likelihood for the specific mention of mental disorders alongside other noncommunicable diseases.^2^ Concurrent with the process of defining the SDG agenda, a parallel global consensus is emerging around ensuring universal health coverage (UHC) as a guiding principle for health care delivery in all countries. With the United Nations General Assembly unanimously adopting the landmark resolution endorsing UHC as a global priority for sustainable development, it is likely that one of the health targets will be the attainment of UHC.^1^

The principles underlying UHC are relatively simple: evidence-based interventions should be adopted by the health care system with the goal of provision to all persons in the population through delivery channels which are prepaid, typically through taxes (but also through insurance in some countries) so that care is mostly free at the point of delivery.^3^ The pooling of risk, such that the rich subsidize the poor and that the healthy subsidize the sick, is at the heart of the financing principles of UHC. There is a strong emphasis on integrated care with much care being delivered through an adequately resourced primary care system, supported by higher levels of care where needed. The substantial resources needed for UHC, particularly difficult to achieve in many low and middle income countries, means that difficult decisions need to be made to identify which interventions will be included, at least in the initial stages, in the package of services. Thus, a progressive realization of UHC is recognized as the most likely course for most countries, both in terms of coverage of the population (starting with the poorest and incrementally increasing till the coverage encompasses the entire population) and in terms of the conditions and interventions.^3^ In the latter case, decisions often guided by the metrics of mortality and cost-effectiveness: thus, the conditions associated with the highest populations burden of mortality and the most cost-effective interventions are prioritised, with coverage for other conditions considered a future goal.

Not surprisingly, interventions for schizophrenia are not attractive when assessed with these metrics, for the condition is relatively rare and accounts for a small share of the global number of deaths; for example, the latest Global Burden of Disease reports just 0.02% of global deaths being attributed to schizophrenia.^4^ Furthermore, the best available interventions are neither curative nor lifesaving, rendering them less attractive when compared with interventions such as antidepressants or antiretrovirals. It is therefore not surprising that of all the mental disorders depression, which is associated with high burden and cost-effective interventions and for which the counter-factual case of the cost of inaction is compelling, has attracted most attention. Yet, this article argues that we need to ensure that a narrow focus on burden and cost-effective interventions must not be allowed to outweigh the enormous health, economic and social hardships, including the significantly increased mortality rates^5^ and some of the worst instances of human rights abuse witnessed in modern times,^6^ in ensuring the inclusion of a basic package of services for schizophrenia. Indeed, the magnitude of the impact of schizophrenia on individuals should be recognized as an equally important driver of prioritisation. The relaxation of a narrow focus on cost-effectiveness in priority-setting has already been seen in the WHO’s recent inclusion of new expensive anticancer drugs in the recommended list of essential drugs, a significant step against the tide of recent years towards privileging population health over an individual’s right to health.^7^

To make the case for the inclusion of care for people with schizophrenia in the basic package of services in UHC, we must address a number of key questions: what interventions should be proposed for scale-up; how should they be delivered, particularly in low resourced settings with few mental health specialists; and what are the barriers to their delivery and how should these be addressed. Fortunately, there is now a robust evidence base which offers a clear path in response to the first 2 questions. While the barriers faced offer a clear path for future research, they have not deterred some countries such as Brazil and China, from rapidly scaling up packages of care within the framework of UHC.

## What Interventions Should Be Selected?

The WHO’s mhGAP guidelines^8^ and the forthcoming third edition of the Disease Control Priorities (DCP3) recommendations^9^ for interventions for schizophrenia consistently show that 2 types of interventions, used in varying combinations tailored to the needs for individual patients, have sufficiently strong evidence backing them to demand inclusion in a scaled up package of services. The first type of intervention is medications, in the form of antipsychotic drugs. Although there are several classes of drugs currently in the market, it seems clear that these mainly differ from one another in terms of their side effects, but are otherwise comparable in terms of their clinical effectiveness (barring the singular exception of clozapine). Thus, the recommendation is to use either typical or atypical drugs, based on availability and a preference for generics, to allow some flexibility to enhance tolerance and adherence. The second type of intervention comprises psychosocial interventions as summarized by the Schizophrenia Patient Outcomes Research Team.^10^ In brief, this group recommended eight interventions: assertive community treatment, supported employment, cognitive behavioral therapy, family-based services, token economy, skills training, psychosocial interventions for alcohol and substance use disorders, and psychosocial interventions for weight management. However, the evidence supporting these approaches is lacking in low and middle income countries (LMIC) and, even in high income countries, few of these interventions have been effectively scaled up. A more pragmatic, from the perspective of acceptability and feasibility, interpretation of psychosocial interventions can be found in the experiences of LMIC innovators, such as programs for community care intervention for people with chronic schizophrenia in India^11^ and those implemented by the NGO Basic Needs as part of its ‘mental health and development’ model in several countries, which have been shown to be associated with improved outcomes, in particular in reduction in levels of disability.^12,13^ The key to these approaches is the selection of relatively low intensity psychosocial intervention components which target patient defined outcomes^14^ and include psycho-education, family support, adherence support, befriending and self-help groups, practical problem solving or social case work (eg, provision of shelter for the homeless) and physical health promotion.^11^

## How Should These Interventions Be Delivered?

The question of how effective interventions should be delivered in low resource settings where there are both structural barriers related to the low availability and inequitable distribution of mental health resources and high levels of impoverishment associated with severe mental disorders, as well as demand barriers related to prevailing explanatory models which interpret these conditions from nonbiomedical perspectives and low awareness of alternative perspectives, has been a major challenge facing mental health care systems in most countries around the world. The experiences of country or regional level programs, demonstration projects by NGOs and clinical trials, have been synthesized in several recent reviews^15–18^ and is a major theme of the DCP3 volume.^9^ In summary, the model of care is a collaborative one, involving a partnership between 4 key players: the patient, family members, a community based nonspecialist worker, and a psychiatrist. Ideally, care is provided within a catchment area based framework and involves relevant social care agencies as required for the individual. The ultimate goals are maximising effective coverage (as reflected both in terms of engagement with the intervention and optimized patient-defined outcomes), reducing human rights violations, mobilizing patient self-help groups, enhancing early detection and generating demand for services. The role of the community based worker are to promote early detection of new cases and of relapse, promote social inclusion, deliver specific psychosocial interventions tailored to the individual’s needs, mobilize social networks, support family members in addressing their own concerns and providing humane care, and support adherence with medications and healthy lifestyles. The roles of the psychiatrist are to diagnose, monitor the course of the illness and drug treatment, supervision of the community health workers, and provision of care in the context of acute emergencies and refractory cases. In addition to outpatient and community care, the role of inpatient care for acute crises and residential care for persons who have limited resources for independent living are key delivery platforms, but ideally these should not be in large psychiatric hospitals.

## Barriers to Scaling Up

Thus, we now know what interventions to deliver and how to do so even in settings with scarce mental health resources. It may appear that all we now need is money to make this happen. However, there remain a number of barriers to maximize the coverage of these interventions. I consider 5 critically important barriers which need to be addressed and propose some promising strategies to address these.

The first is the difficulty in scaling up the task sharing of the care of schizophrenia to community based nonspecialist workers. While such recommendations are already being advocated in a number of country policies, for example in India’s new National Mental Health Policy, their actual deployment at scale remains a challenge due to a number of barriers in scaling up the task-sharing of mental health care.^19^ The most formidable barriers are those of creating and sustaining a vast number of new posts, albeit at the relatively low-cost bottom end of the health workforce pyramid, in the routine health care system and ensuring quality in acquiring and maintaining competencies in a scaled up program. One major factor hindering task-sharing is the resistance, and even hostility, of some sections of the mental health professional communities towards this strategy. The growing acceptance of community based workers to address other chronic and disabling conditions such as cardio-metabolic and neurodegenerative disorders^20^ provides a unique opportunity to integrate the care of schizophrenia within the broader role of a community based chronic disease worker.

The second barrier is the very low numbers of psychiatrists in many parts of the world which make it difficult to implement a collaborative care model relying on a psychiatrist. The ability to share tasks of diagnosis and drug treatments to nonspecialized workers have not been documented with confidence, not only because of the skills and expertise involved but also because of regulatory restrictions (for eg, in some countries only medically qualified mental health professionals are allowed to prescribe antipsychotic drugs or these drugs are only available through specialized facilities). These challenges are further compounded by the reluctance of primary care physicians to take on roles related to mental health care, in particular for severe mental disorders. The deployment of midlevel mental health workers such as psychiatric nurse practitioners and using telemedicine to improve access to specialists are promising strategies to address this barrier.^21^

The third barrier is the considerable delay in the diagnosis of schizophrenia, particularly in remote and rural communities, both due to lack of access to skilled providers and due to the different explanatory models prevailing in these communities. A typical consequence is that diagnoses may never be made or only be made during acute crises. These challenges are being addressed through novel population based approaches for identifying psychoses as a broad clinical syndrome and engaging a wide variety of key gatekeepers, including traditional medical practitioners and religious leaders in the community.^22^ The key goal is a pragmatic model for early detection to reduce the duration of untreated psychosis.

The fourth barrier is the lack of evidence-based health system guidance on the management of acute emergencies associated with schizophrenia, in particular acute psychotic episodes, in routine casualty services by nonspecialist health workers. A recent systematic review revealed the complete absence of empirical evidence to guide nonspecialist health workers, often the first point of contact for psychiatric emergencies, in the management of acute crises.^23^ There is a need for systematic guidelines, based on practice based evidence, for primary and general health care staff and other key stakeholders who may encounter such situations, such as police and social welfare personnel, on the care of affected individuals. Such guidelines must be consistent with the global human rights conventions which put emphasis on supported decision making and minimal use of involuntary treatments.

The final barrier is the lack of agency for people with schizophrenia and their families to demand care which is aligned with their own priorities, and the rapidly changing social circumstances consequent upon urbanization and smaller household sizes which are narrowing the opportunities for productive engagement of the affected person in livelihood activities and reducing the capacity of families to care for affected relatives. The empowerment of people with schizophrenia and their families through creation of self-help groups^24,25^ and an explicit recognition of the need to build livelihood skills^12^ are some examples of strategies to address this barrier.

## Integrating Psychoses Care in UHC: Country Case Studies

A number of countries, mostly in the middle income category, have embarked on ambitious programs to expand coverage of care for people with schizophrenia. Two notable examples stand out: China and Brazil.

In China, the National Continuing Management and Intervention Program for Psychoses (also referred to as the 686 program because the project received its first financial allotment of 6.86 million Renminbi, equivalent to $829 000, in 2004 when it was launched), seeks to integrate hospital and community based care for psychoses. It was the first noncommunicable disease program in the country to be included in the national public health agenda. The program began with 60 demonstration sites, half each in rural and urban areas, covering a total population of 43 million. The components of the intervention included patient registration and initial assessment, free medication and regular follow-up in the community, management of community emergencies, and free emergency hospitalization. The project identified and treated many patients who had previously been locked up in their homes by family members. By 2014, the program has reached most parts of the country and over 4 million patients have been registered. An internal evaluation in 2011 reported that the rate of clinical “stability” (ie, without relapse of acute symptoms) had risen from 67% to 90%.^26^ A large number of personnel were trained including 10000 psychiatrists, accounting for half of all psychiatrists in China, and the mental health service team nationwide has been enlarged 7 fold in the past 7 years.

In Brazil, a nationwide mental health reform effort was initiated as an integral component of the National Public Health System (called the SUS) created by the 1988 Constitution, whose objective is to guarantee access to a full range of health care services to the entire population. The key components of the mental health reform was the reduction of beds in psychiatric hospitals and its replacement by a community based system capable of addressing both acute crises and continuing care. Since the implementation of the program, the number of beds in psychiatric hospitals has dropped by more than half, with a focus on removal of beds in institutions with poor quality of care and large size. As one indicator, small size hospitals (up to 160 beds) which accounted for 24% of the beds in 2002, represented almost half of the total of the beds in 2010. The main platform of the community care delivery was the Center of Psychosocial Care (CAPS), whose coverage has gradually increased since its launch in the late 1980s to 66% of the country’s population in 2010. One of the striking features of SUS is the strengthening and expansion of primary health care, through the establishment of family health teams (of the Family Health Program) comprising community health workers. Since 2002, the CAPS was designed to coordinate its delivery system with primary care. In 2008, special teams for supporting primary care were created, aimed at strengthening the link between primary care teams and some health domains, such as mental health. The entire network of mental health services created under this reform process include: CAPS, primary care teams, residential services, mental health services in general hospitals, social cooperatives and work initiatives, cultural initiatives, street offices for drug consumers, and clubs (community leisure centers).^27^

While both these countries demonstrate the feasibility of integrating psychoses care within the framework of UHC through a combination of political will, adequate financial resources and attention to technical aspects of the implementation, 2 caveats need to be borne in mind: first, both of these are upper middle-income countries with rapidly expanding economies and thus substantially greater resources available for such programs compared with many other developing countries; and second, there are no independent evaluations of these programs.

## How Much Will It Cost to Scale-up?

A simple comparison of the cost of a community based vs hospital-based service model has been carried out as part of a WHO cost-effectiveness analysis for schizophrenia and bipolar affective disorder. For schizophrenia, the costs of the hospital-based service model exceeded those of the community based service model by 33%–50%, reflecting greater use of resource-intensive services, such as acute and long-term psychiatric inpatient care.^28,29^ This approach to service costing has been applied more recently to the subnational context of scaling up mental health services in LMICs, as part of the PRogramme for Improving Mental health carE study being conducted at the district level in Ethiopia, India, Nepal, South Africa, and Uganda.^30^ The results indicated that, starting from a generally very low base of service coverage and expenditure, the cost of scaled-up provision in nonspecialist health care settings of an evidence-based package of care that included psychosis, depression, alcohol use disorders and, in some countries, epilepsy, range from US$ 0.25–0.70 per capita in 4 out of the 5 districts assessed. For a district with a total population of 1 million persons, therefore, an annual outlay of US$250,000–US$700,000 would be required to reach specified target coverage levels. The outlier is South Africa, where the prevailing price and quantity of health care service inputs are higher. The cost per capita of delivering the specified care package at target coverage levels in the South African district approaches US$2.50 per capita; this is higher than in the other countries but relatively low in the context of current health spending levels in South Africa.

New analytic work of the DCP3 consortium has shown that adequate financial investments for schizophrenia are not meagre; for example, when specialist service need is factored in for a proportion of cases, the cost per treated case in India is estimated to be $177 per year (not an insignificant sum given that this is 5 times more than a case of depression and a large proportion of the population lives on less than a dollar a day) or equivalent to $0.39 per head of population. However, as recent empirical work has shown, there is the potential for such investments to make significant economic contributions to society, for example through increased earnings, increased household income and reduced burden on caregivers who are able to undertake more economically productive activities.^31^ Since much of this cost is currently paid for out-of-pocket by households, moving to a situation where the large majority of cases have all their care financed publicly implies a large injection of new resources by government. Such an increase in service and financial coverage for schizophrenia treatment in India, however, would have a clear pro-poor effect: 30% of the total insurance value is bestowed on the poorest quintile of the population, compared with 10% for the richest quintile.^9^ Whereas middle income countries should be able to allocate resources from their own tax funded revenues to these expanded programs, is imperative for rich countries to specifically enhance the pitifully meagre allocations of development assistance to mental health, currently at less than 1% of the total development assistance to health.^32^

Relocating services and resources away from long-stay mental hospitals toward nonspecialized health settings is a key financing issue for mental health systems in many countries. Efforts to change the balance of mental health care are often hindered by a lack of appropriate transitional funding. Transitional or dual funding will be required over a period of time to build up appropriate community based services before residents of long-term institutions can be relocated. It is crucial to present an evidence-based case for relocating the locus of care, not only on the grounds of equity, human rights, and user satisfaction, but also on the grounds of financial feasibility over a defined transitional period.

## Conclusions

There is a robust evidence base testifying to the effectiveness of drug and psychosocial interventions for people with schizophrenia and to the feasibility, acceptability and cost-effectiveness of the delivery of these interventions through a collaborative care model in low resource settings. While there is a need for continuing investments in implementation research seeking to address the barriers to scale-up, the experiences of upper middle income countries like Brazil and China show that political commitment, allocation of transitional financial resources, a commitment to an integrated approach with a strong role for community based institutions and providers, and a progressive realization of coverage (both in terms of area and range of services), are the key ingredients for scale up of services for schizophrenia. The growing momentum towards a global compact on UHC, alongside the acknowledgment of the urgency and importance of a comprehensive mental health action plan^33^ offers a unique opportunity for a substantial scale-up of evidence-based interventions and packages of care for a range of mental disorders in all countries. Starting with schizophrenia, arguably the most visible and stigmatized mental disorder in any community, not only invokes the principle of the right to care for the most disabled and the most disadvantaged, but also frames a path towards the ultimate universalization of care for the full range of mental disorders.

## Funding

Wellcome Trust Senior Research Fellowship in Clinical Science (091834/Z/10/Z to V.P.); the UK Aid which funds PRIME.
